# Medical Students’ Acceptance of Digital Entrustable Professional Activities: Results of a Cohort Study

**DOI:** 10.2196/87605

**Published:** 2026-05-04

**Authors:** Maximilian Domann, Constanze Richters, Matthias J Witti, Matthias Stadler

**Affiliations:** 1 Institute of Medical Education, LMU University Hospital, LMU Munich, Pettenkoferstr. 8A, Munich, Bavaria, 80336, Germany, 49 089 4400 ext 57360

**Keywords:** medical education, entrustable professional activities, simulation, technology acceptance model, digital learning, undergraduate medical education, EPA, TAM

## Abstract

**Background:**

Digital entrustable professional activities (EPAs) in simulated environments may accelerate competency acquisition, but adoption depends on learner acceptance. The Technology Acceptance Model (TAM) posits that perceived usefulness (PU) and perceived ease of use (PEU) shape attitudes (AT) and, in turn, behavioral intention (BI).

**Objective:**

This study aimed to examine medical students’ acceptance of digital EPAs and to test the hypothesized TAM relationships among PU, PEU, AT, and BI.

**Methods:**

Clinical-phase medical students at Ludwig Maximilian University of Munich completed a TAM-based survey (7-point Likert scales) after reading a canonical analog EPA and its digital counterpart. Confirmatory analyses comprised bivariate correlations and hierarchical regressions testing TAM paths. Exploratory analyses comprised paired-sample two-tailed *t* tests comparing analog versus digital ratings and path modeling to evaluate global TAM fit.

**Results:**

Between 70 and 72 medical students provided complete, usable responses, depending on the construct. Mean ratings were favorable (≈5/7). Internal consistency was acceptable (*ω*=.67–.80). Within the digital EPAs, PU strongly predicted AT (*β*=.59; *P*<.001), and AT predicted BI (*β*=.58; *P*<.001). For the analog EPAs, PU (*β*=.54; *P*<.001) and PEU (*β*=.28; *P*=.005) predicted AT; both AT (*β*=.42; *P*<.001) and PU (*β*=.36; *P*=.002) predicted BI. Attitudes were modestly higher for analog versus digital (M=5.18 vs 4.87; *t*_71_=−2.50, *d*=−0.30; *P*=.02), but PU, PEU, and BI did not differ significantly. The path models indicated excellent fit for both formats (comparative fit index=1; root mean square error of approximation=0; standardized root mean square residual ≤.01).

**Conclusions:**

Students reported high acceptance of digital EPAs. Acceptance was driven primarily by PU (via AT), whereas PEU contributed to AT only for analog EPAs. Implementation should emphasize demonstrable educational value and cultivate positive attitudes; subsequent work should link acceptance to actual use and learning outcomes.

## Introduction

Entrustable professional activities (EPAs) have reshaped medical education by shifting the focus toward competency-based learning. EPAs break down complex clinical skills into manageable units, enabling learners to acquire proficiency progressively while allowing teaching staff to structure supervision and maximize learning outcomes [1,2]. In competency-oriented curricula, responsibilities and levels of autonomy are made explicit and can be increased as competence grows, culminating in independent performance with appropriate oversight [3,3-7]. Such transparency benefits students, educators, and institutions by aligning expectations and clarifying which tasks can be performed at which level of supervision [7,8].

In parallel, simulation-based training has become integral to contemporary medical education. Simulations create realistic, interactive environments in which students can safely practice essential cognitive and motor skills without risk to patients. Computer-based simulations in particular provide dynamic representations of clinical scenarios and have demonstrated benefits for decision-making and procedural learning in higher education [7,9-12]. In domains such as surgery, where theoretical knowledge must be combined with hands-on technique, simulation-based training has shown promise for accelerating skill acquisition and transfer to clinical performance [12]. Recent reviews further highlight that advances in digital technologies, including immersive simulations and data-driven feedback systems, are increasingly shaping how simulation-based training is designed and implemented in medical education [13].

Despite the established value of both EPAs and simulation-based training, their systematic integration into digital EPAs remains underexplored. We use the term “digital EPAs” to denote entrustable tasks instantiated in simulated or virtual environments so that learners can rehearse the same competencies before encountering them in real clinical care. Digital EPAs could enhance learning efficiency, enable standardized exposure to complex scenarios, deliver immediate and scalable feedback, and capture rich performance data to inform programmatic assessment. Recent evidence from virtual simulation environments further suggests that such digital formats may also enhance learners’ motivation and perceived value of training, thereby supporting acceptance alongside learning outcomes [14]. Beyond medical education, recent work across clinical professions highlights that digital health competencies are increasingly expected in clinical practice, yet remain inconsistently embedded in formal training and competency frameworks, underscoring the need for structured educational approaches [15]. At the same time, digital EPAs may introduce new barriers, including usability burdens, cognitive overload, or mismatches with learners’ expectations and existing curricular workflows. These competing forces make it insufficient to assume that established benefits of simulation or EPAs will automatically translate to digital EPAs. Rather, acceptance by end users must be established empirically before large-scale implementation is warranted [9,12,16].

To examine technology adoption in this context, the Technology Acceptance Model (TAM) provides a robust explanatory framework. TAM posits two central beliefs—perceived usefulness (PU) and perceived ease of use (PEU)—that shape attitudes (AT) toward a technology and, in turn, behavioral intention (BI) to use it (and ultimately actual usage) [17,18]. PU captures the belief that a system will enhance performance; PEU reflects the effort required to operate it. Together, these constructs explain why learners adopt educational technologies and provide a practical framework to identify leverage points for implementation in medical education.

In medical education, where novel digital tools continue to proliferate, applying TAM can yield actionable insights into which features matter most for learner uptake and sustained use. A recent systematic review confirms the continued relevance of the TAM in medical education, demonstrating its applicability across a broad range of digital learning technologies and educational contexts [19]. Prior work applying TAM in educational contexts has demonstrated that PU and PEU can meaningfully predict AT and BI toward a wide range of learning technologies [20,21]. However, the specific TAM predictors of acceptance for digital EPAs have not yet been characterized. While prior methodological work has outlined approaches for developing digital health–related EPAs and defining their structure and content, empirical evidence on learners’ acceptance of such digital EPA formats remains scarce [16].

By operationalizing TAM constructs for this setting and testing their associations with behavioral intention among clinical-phase medical students, this study seeks to provide evidence to guide the design of learner-centered digital EPAs and inform decisions about their integration into medical curricula. By focusing on learners in the clinical phase, where competency acquisition and supervised autonomy are most salient, this study addresses a critical evidence gap at the intersection of EPAs, simulation, and technology acceptance. The findings aim to inform the design and implementation of digital EPA curricula and to support educators and developers in aligning functionality and user experience with the factors that drive adoption.

Based on the predictions of the TAM, we hypothesize that:

- Hypothesis 1: the more useful medical students find digital EPAs in medical simulations, the higher their behavioral intention to use this technology as part of their studies will be
- Hypothesis 2: the easier medical students find the use of digital EPAs in medical simulations to be, the greater their behavioral intention to use this technology will be
- Hypothesis 3: the more positive the attitude of medical students toward digital EPAs in medical simulations is, the higher their behavioral intention to use this technology will be

In addition, we will also test these hypotheses for analog EPAs to exploratively compare the fit of the TAM for analog EPAs to that of digital EPAs.

## Methods

### Overview

The methodology closely aligns with the Research Protocol, which describes it in more detail [17]. Further, the digital EPA condition was assessed using a standardized narrative description of a hypothetical digital EPA rather than interaction with a functional software system, as no such system was available at the time of the study.

### Participants and Recruitment

#### Overview

This cohort study targeted medical students in the clinical phase of their studies at Ludwig Maximilian University (LMU) Munich. This population was optimally suited for the application of digital EPAs, as the clinical phase centers on acquiring specific clinical skills. The survey link was distributed through major student email lists (eg, the “Module 6” distribution list), ensuring that only students in the clinical phase received the invitation. Participation was voluntary, and students could complete the questionnaire at their convenience; overall, no incentives were offered.

#### Inclusion Criteria

Participants had to be at least in their third year of medical school (having completed the preclinical foundational courses in anatomy, physiology, biochemistry, etc) and must have passed the first medical licensing examination. This ensured that all respondents had a sufficient clinical and educational background to appraise EPAs in context.

#### Exclusion Criteria

Students needed a sufficient command of the German language to understand the study materials and survey, since the questionnaire and EPA descriptions were provided in German.

### Survey Instrument and Hypotheses

We developed a web-based questionnaire using SoSciSurvey based on the TAM, adapting an existing one from Abdul Ghani et al [18] to the context of digital EPAs [19].

The survey included items (rated on 7-point Likert-type scales) to measure four TAM constructs: PU, PEU, AT, and BI. Each construct was assessed with a multiitem scale, and reliability (internal consistency) was measured with McDonald ω. Prior to fielding the survey, the questionnaire was reviewed for clarity and technical functionality and was internally pilot-tested to ensure usability and correct item presentation. Data collection took place between May 2025 and June 2025.

### Study Procedure and Materials

Because EPAs (especially in digital form) are a relatively unfamiliar concept for students, we provided participants with a concrete example of an EPA and its digital counterpart before they answered the survey. Specifically, participants were shown a description of a sample analog EPA (a clinical task with defined supervision levels) and a description of the same task implemented as a hypothetical digital EPA. These materials were developed based on the guidelines by Cate and Taylor [2] for formulating EPAs. The digital EPA was presented conceptually (in narrative text form) since no fully functional digital platform was available; students were asked to imagine using a system that would allow them to perform the EPA in a simulated digital environment. All materials, as well as the full descriptions of the example EPA (analog version and conceptual digital version), are available in the project’s Open Science Framework repository [22] as well as MultimediaAppendices 1^4^. Both German and English versions of these descriptions were provided (the survey itself was administered in German).

After reviewing the EPA descriptions, participants proceeded to the TAM questionnaire. They were instructed to respond to the TAM items twice: once reflecting on the analog EPA scenario and once for the digital EPA scenario. In other words, for each TAM construct (PU, PEU, AT, and BI), students provided separate ratings regarding (1) the traditional analog EPA and (2) the digital EPA. This within-subjects design allowed direct comparison of acceptance between the analog and digital formats.

### Statistical Analysis

All analyses were conducted using the jamovi software [20]. We first computed descriptive statistics for each TAM construct (mean and SD) and evaluated internal consistency (McDonald ω) for each multi-item scale. We examined bivariate correlations among all TAM constructs (PU, PEU, AT, and BI) separately for the digital EPA ratings and the analog EPA ratings.

To test our hypotheses about the determinants of attitude and intention, we performed hierarchical multiple regressions. For attitude (AT) as the outcome, we entered PEU in the first model (to assess its effect alone) and then added PU in the second model, for both digital and analog conditions. This allowed us to see whether PU provided additional explanatory power for attitude beyond PEU. Similarly, for behavioral intention (BI) as the outcome, we first entered attitude alone (Model 1), then added PU in a second model, analyzing digital and analog conditions separately. In each case, we report standardized regression coefficients (β), *P* values, the proportion of variance explained (R²), and the change in R² for the stepwise addition.

We supplemented the regression analyses with path modeling to simultaneously evaluate the TAM relationships (PEU → AT, PU → AT, AT → BI, and direct PU → BI) and to compare model fit for the digital and analog data. Model fit was assessed with the comparative fit index (CFI), Tucker-Lewis Index (TLI), root mean square error of approximation (RMSEA), and standardized root mean square residual.

Finally, although not specified in the original protocol, we conducted exploratory paired-sample 2-tailed *t* tests to directly compare students’ mean ratings for the analog EPA versus the digital EPA on each TAM construct. This was done to gage any overall preference for one format over the other (eg, whether attitudes toward the analog EPA were significantly different from attitudes toward the digital EPA, etc). Effect sizes for mean differences were calculated using Cohen *d*.

All hypothesis tests used a significance level of *α*=.05 (2-tailed). The target sample size (n=68) was determined a priori via power analysis (G*Power 3.1) for detecting moderate effect sizes with power 0.80 at *α*=.05 in regression models [23].

### CHERRIES Checklist

The online survey was conducted and reported in accordance with the CHERRIES (Checklist for Reporting Results of Internet E-Surveys) checklist. The questionnaire was distributed over multiple pages with a limited number of items per page. Participants were able to review and modify their responses before final submission. No randomization or adaptive questioning was applied. To prevent multiple entries from the same individual, the survey platform used standard technical measures (eg, cookies and IP-based restrictions). No personally identifiable information was collected. View rates and participation rates could not be calculated, as access logs of unique visitors were not available.

### Ethical Considerations

This study was approved by the ethics committee of the LMU University Hospital, Munich (project No. 23‐0577). Before accessing the questionnaire, participants were presented with an electronic informed consent page describing the study purpose, voluntary participation, approximate survey duration, and data handling procedures. No personally identifiable information was collected, and all data were stored on secure servers in accordance with institutional data protection regulations. Participants received no compensation for their participation.

## Results

### Overview

We first examined students’ overall evaluations of analog and digital EPAs to establish baseline acceptance and scale reliability. Depending on the construct, between 70 and 72 students provided complete, usable responses (the exact *n* varies slightly because some participants left a few items blank for certain scales). All analyses below reflect the data from those who provided complete responses for the given measures.

### Descriptive Statistics and Reliability

Overall, students’ evaluations of both the digital EPA and the analog EPA were positive. Mean scores on the 7-point scales were around 5 for all TAM constructs (Table 1). For the digital EPA, the mean scores (with SDs) were 4.94 (SD 1.10) for perceived usefulness, 4.94 (SD 1.21) for perceived ease of use, 4.87 (SD 1.13) for attitude, and 4.85 (SD 1.05) for behavioral intention. Analog EPAs were rated slightly higher on average, with means ranging from 4.98 to 5.18. All constructs demonstrated acceptable internal consistency, with McDonald ω coefficients between .67 and .80 across the scales. The lower coefficients (particularly ω≈.67) are plausible in this early-stage application of an adapted TAM instrument to a novel and partly hypothetical learning concept, where item interpretations may still vary across respondents. Moreover, the relatively small number of items per construct and the homogeneous sample can attenuate internal consistency estimates.

**Table 1.** Descriptive statistics and internal consistency of Technology Acceptance Model constructs (n=70‐72).

Construct	Digital EPAs^a^, mean (SD)	n	Digital ω	Analog EPAs, mean (SD)	n	Analog ω
Perceived usefulness (PU)	4.94 (1.10)	72	.75	5.17 (1.09)	72	.80
Perceived ease of use (PEU)	4.94 (1.21)	70	.76	5.02 (0.99)	70	.67
Attitude (AT)	4.87 (1.13)	72	.79	5.18 (0.97)	72	.73
Behavioral intention (BI)	4.85 (1.05)	72	.74	4.98 (1.02)	72	.72

a EPA: entrustable professional activity.

Overall, these descriptive results indicate generally favorable evaluations of both analog and digital EPAs, with mean ratings close to the upper end of the scale and acceptable internal consistency across constructs.

### Exploratory Comparison of Analog vs Digital

To explore whether students evaluated analog and digital EPAs differently overall, we conducted paired-sample 2-tailed *t* tests comparing ratings across formats. These exploratory analyses (which were not specified in the study protocol) revealed that attitudes toward the analog EPA were slightly but significantly more positive than attitudes toward the digital EPA (*t*_71_=–2.50, *d*=–0.30; *P*=.02). In contrast, there were no statistically significant differences between analog and digital formats in perceived usefulness (*t*_71_=–1.46; *P*=.15), perceived ease of use (*t*_69_=–0.39; *P*=.70), or behavioral intention (*t*_71_=–0.73; *P*=.47). Thus, apart from a small difference in attitude favoring the analog EPA, students’ evaluations of usefulness, ease of use, and behavioral intention did not differ substantially between formats.

### Correlations Among TAM Constructs

Before testing the hypothesized TAM relationships in regression analyses, we examined bivariate correlations among all TAM constructs for both digital and analog EPAs. Bivariate correlations between all pairs of constructs are presented in Table 2 for the digital and analog versions, respectively. As expected, the TAM variables were moderately to strongly intercorrelated. For digital EPAs (Table 2), perceived usefulness had a strong positive correlation with attitude (*r*=.64; *P*<.001), and attitude in turn was strongly correlated with behavioral intention (*r*=.70; *P*<.001). Perceived ease of use showed moderate positive correlations with both perceived usefulness (*r*=.45; *P*<.001) and attitude (*r*=.55; *P*<.001). For analog EPAs (Table 2), a very similar pattern emerged: perceived usefulness correlated strongly with attitude (*r*=.66; *P*<.001), and attitude correlated strongly with behavioral intention (*r*=.65; *P*<.001). The correlation between perceived ease of use and perceived usefulness was of moderate size (*r*=.41; *P*<.001), as was the correlation between perceived ease of use and attitude (*r*=.49; *P*<.001). Overall, the correlation patterns were consistent with the TAM and justified proceeding to regression and path analyses.

**Table 2.** Correlations among Technology Acceptance Model constructs for digital entrustable professional activities (n=70-72)^a^.

Variable	Perceived ease of use	Perceived usefulness	Attitude	Behavioral intention
Perceived ease of use	—	.41	.49	.46
Perceived usefulness	.45	—	.66	.61
Attitude	.55	.64	—	.65
Behavioral intention	.50	.48	.70	—

a Values below the diagonal are for analog entrustable professional activities, values above the diagonal are for digital entrustable professional activities. All correlations *P*<.001.

### Predictors of AT

We next examined which TAM variables predicted students’ attitudes toward EPAs using hierarchical regression analyses. For digital EPAs, perceived ease of use alone (Model 1) explained 31% of the variance in attitude, but in this model PEU’s effect was not statistically significant (*β*=.14; *P*=.29). When perceived usefulness was added (Model 2), the model’s explanatory power increased significantly (ΔR²=.17, total R²=.48; *P*<.001). In the final model for digital EPAs, PU emerged as a strong positive predictor of attitude (*β*=.59; *P*<.001), whereas PEU remained nonsignificant (*β*=.14; *P*=.29).

For analog EPAs, perceived usefulness alone accounted for 44% of the variance in attitude (Model 1, *β*=.68; *P*<.001). Adding perceived ease of use (Model 2) produced a small but significant increase in explained variance (ΔR²=.06, total R²=.51; *P*=.005). In the final analog model, both predictors had significant effects: PU (*β*=.54; *P*<.001) and PEU (*β*=.28; *P*=.01) each independently contributed to a positive attitude. In summary, perceived usefulness emerged as the dominant predictor of attitude in both formats, whereas perceived ease of use contributed additionally only in the analog condition.

### Predictors of BI

Subsequently, we analyzed predictors of behavioral intention to use EPAs. For digital EPAs, students’ attitude toward the digital EPA was by far the dominant predictor of their behavioral intention. Attitude alone explained 48% of the variance in BI (Model 1, *β*=.58; *P*<.001). Adding perceived usefulness did not significantly improve the model (ΔR²=.01; *P*=.40), and in the full model attitude remained the only significant predictor of BI (*β*=.58; *P*<.001, while PU’s direct effect was *β*=.09; *P*=.17, not significant). This indicates that for digital EPAs, behavioral intention was primarily driven by students’ attitude, and any influence of perceived usefulness operated through attitude rather than as a separate direct effect.

For analog EPAs, attitude was also a strong predictor of BI, accounting for 43% of variance on its own (Model 1, *β*=.42; *P*<.001). However, in contrast to the digital case, adding perceived usefulness yielded a significant improvement (ΔR²=.07; *P*=.002), bringing the total R² to .51 for Model 2. In the final analog model, both attitude (*β*=.42; *P*<.001) and PU (*β*=.36; *P*=.002) had significant independent effects on behavioral intention. Thus, for analog EPAs, students’ intention to use was jointly determined by their attitude and their perceived usefulness of the EPA.

### Path Modeling

Finally, to evaluate the overall fit of the hypothesized TAM structure, we conducted path modeling for both digital and analog EPAs. We specified a model in which PEU and PU both load onto AT (with direct paths PEU→AT and PU→AT), AT loads onto BI (AT→BI), and PU also has a direct path to BI. This corresponds to the classic TAM with attitude as a mediator and a possible direct effect of usefulness on intention. The model was estimated separately for the digital EPA ratings and the analog EPA ratings. Model fit was excellent for both the digital model and the analog model: CFI=1, TLI=1.05 for both; RMSEA=0; standardized root mean square residual=0.004 (digital) and 0.002 (analog).

These fit indices generally indicate that the hypothesized TAM relationships almost perfectly reproduced the observed covariance patterns in our data. We interpret these findings cautiously since global indices such as CFI or RMSEA may appear near perfect even in modest samples when models are small and comparatively constrained. Accordingly, we focus primarily on the pattern and magnitude of the path estimates, which converged with the hierarchical regression findings.

The path estimates were consistent with the regression findings. In the digital EPA model, perceived usefulness had a significant positive effect on attitude, and attitude had a strong positive effect on behavioral intention. The direct path from PU to BI was not significant (in line with our earlier result that attitude mediated the effect of PU). In the analog EPA model, both perceived usefulness and perceived ease of use significantly influenced attitude, and both attitude and PU significantly influenced behavioral intention. In both models, the vast majority of the explainable variance in BI was accounted for (which is reflected in the high *R*² values of ~0.5 in the regressions and the excellent fit indices here). Overall, the path models corroborated the regression findings and supported the applicability of the TAM structure to both EPA formats.

## Discussion

### Principal Findings

This study examined medical students’ acceptance of digital EPAs within simulated learning environments, guided by the TAM. Several important findings emerged. First, perceived usefulness was the strongest predictor of students’ attitudes toward using digital EPAs, and attitude in turn was the primary driver of behavioral intention. For digital EPAs, once attitude was taken into account, perceived usefulness no longer had a direct effect on behavioral intention, suggesting that the influence of usefulness operated mainly by shaping favorable attitudes.

Second, while perceived ease of use predicted attitudes toward analog EPAs, its contribution was not statistically significant for digital EPAs. This pattern may reflect that usability-related considerations were less salient in the hypothetical digital condition and should therefore be interpreted cautiously.

Third, students’ attitudes toward the analog EPA were slightly more favorable than their attitudes toward the digital EPA, although the mean scores for both formats were high overall (both approaching 5 on a 7-point scale). Finally, path modeling confirmed an excellent fit for the hypothesized TAM pathways in both conditions, underscoring the robustness of the TAM framework in this new educational context.

Together, these findings highlight that medical students are generally open to the concept of digital EPAs, but their acceptance seems to be primarily associated with perceived educational value in this study context, while usability considerations may become more salient once learners interact with a functional system. In practical terms, this pattern suggests that effective implementation of digital EPAs will require making the educational benefits transparent and directly aligned with students’ learning goals, rather than focusing exclusively on technical usability or novelty.

### Comparison With Prior Work

The dominance of perceived usefulness as a predictor of attitudes and intentions is consistent with a large body of TAM research in educational technology. Similar patterns have been reported in studies of simulation-based learning, virtual reality training, and digital assessment tools in medical education, where perceived educational value consistently outweighs usability considerations during early adoption phases [9,12,13]. Prior studies on e-learning platforms and immersive digital assessment tools likewise show that perceived usefulness is often a stronger driver of intention than perceived ease of use [16, 21]. Our findings extend this evidence to the novel domain of digital EPAs. While ease of use is usually considered a critical factor when learners first encounter new technologies, its weaker role in our data may reflect the nature of our intervention: the digital EPA was presented conceptually rather than as a hands-on system. This aligns with recent findings from virtual simulation research suggesting that learners’ initial acceptance is primarily driven by perceived relevance and learning value when interaction with a fully functional system is not yet possible [14].

We also observed that attitudes toward the analog EPA were slightly more positive than attitudes toward the digital EPA. This echoes previous observations that learners often exhibit a preference for traditional or familiar tools when a novel digital innovation is first introduced. This kind of “familiarity advantage” may explain why analog EPAs scored marginally higher in our sample. Importantly, however, the differences we found were small, and overall attitudes toward the digital EPA were still quite favorable. This suggests that resistance to this form of digital innovation is not deep-seated and may diminish with increased exposure. Such familiarity effects have also been described in competency-based medical education, where learners initially favor established assessment formats before developing trust in novel digital implementations [15].

Interestingly, in the analog condition, perceived ease of use significantly predicted attitude in addition to perceived usefulness (both contributed to higher attitude in the regression model). This aligns with TAM’s original formulation, in which both usability and usefulness shape attitudes. In the analog context, “ease of use” rather refers to the clarity, structure, and manageability of the EPA tasks (since no technology interface is involved). Students who perceive the analog EPA process as straightforward and not burdensome are more likely to develop a positive attitude toward it. For the digital EPA, by contrast, usefulness alone was sufficient to explain attitude, which may indicate that students either took ease of use for granted in a hypothetical digital system or did not consider it salient without actual experience of the interface.

Our findings also reinforce theoretical assumptions about the mediating role of attitude in technology acceptance. In both the analog and digital models, behavioral intention was strongly predicted by attitude, whereas any direct effects of perceived usefulness on intention became negligible once attitude was accounted for. Interventions to foster acceptance should therefore not only demonstrate the usefulness of a digital EPA (eg, by showcasing how it improves learning outcomes or efficiency), but also aim to cultivate positive attitudes, for example, by providing early positive experiences, highlighting success stories or endorsements from peers, and linking the tool to students’ professional identity and values.

### Strengths and Limitations

This study offers several strengths. It represents one of the first empirical investigations of medical students’ acceptance of digital EPAs, applying the well-established TAM in this context. We obtained satisfactory reliability for all TAM scales, and by using both regression analyses and path modeling, we provided converging evidence for the robustness of the findings. Additionally, by having students consider both a traditional analog EPA and a hypothetical digital EPA, the study provides novel insights into the similarities and differences in what drives acceptance across these formats. This within-subject comparison strengthens our conclusions about the unique contribution of ease of use in the analog format and the primary importance of usefulness in the digital format.

Nevertheless, certain limitations must be acknowledged. First, the sample was drawn from a single medical school, which may limit the generalizability of the results. Cultural, institutional, and curricular factors can influence technology acceptance. For example, the degree to which EPAs and simulation-based learning are already embedded in a curriculum, and more generally, the availability of digital infrastructure as well as institutional attitudes toward educational innovation may shape students’ baseline expectations and acceptance of digital EPAs. Acceptance patterns observed at LMU Munich may therefore differ from those at institutions with less exposure to competency-based curricula or fewer simulation resources. On the other hand, focusing on one institution provided a relatively homogeneous context (all students shared a similar curriculum and culture), which reduced extraneous variance and allowed a clearer interpretation of the TAM relationships. Future research should replicate these findings in larger and more diverse multicenter cohorts to test their robustness across different educational settings.

Second, the study relied on self-report questionnaires for both predictors and outcomes. While surveys are indispensable for capturing perceptions and intentions (and are the standard approach in early-stage TAM research), they do not directly measure actual usage behavior. We cannot be certain that what students *say* they intend to do would translate into real adoption of a digital EPA if one were available. That said, self-report measures of TAM constructs have been widely validated and are valuable for establishing a baseline of acceptance before a technology is fully implemented. Future studies should complement self-reported acceptance data with objective usage analytics and measures of learning outcomes once digital EPA platforms are introduced. This would help determine whether high perceived acceptance indeed leads to sustained engagement and educational benefits.

Third, the “digital EPA” in this study was presented only conceptually rather than as a fully operational system. Students read a description and were asked to imagine using the digital EPA, instead of interacting with a real software application. This design choice was a practical necessity (since no such software existed yet) and allowed us to gage baseline acceptance before investing in development. However, it likely reduced the salience of some usability factors and may have emphasized perceived usefulness. Accordingly, our findings pertain primarily to acceptance of the concept of a digital EPA. In addition, the conceptual and relatively constrained nature of the model, together with the modest sample size, may have contributed to the very high global fit indices observed in the path models. Such near-perfect fit values are not uncommon in simple, theory-driven models and should therefore be interpreted with caution, with greater emphasis placed on the consistency and plausibility of the estimated path coefficients. The relatively small sample size further increases the risk that effect sizes and correlations may be overestimated or appear more stable than they would in larger samples. It is therefore possible that some statistically significant associations observed in this study would be attenuated or no longer reach significance in larger, more heterogeneous cohorts. Replication with substantially larger samples is necessary to assess the robustness of the observed relationships. Once a functional digital EPA platform is developed, future research should assess actual user experience, including any usability hurdles, and examine how these real-world factors might alter the acceptance model. It is possible that ease of use will play a larger role when students can directly experience the interface, features, and potential frustrations of a digital EPA tool.

Finally, the cross-sectional design of this study limits our ability to draw causal conclusions or to observe changes over time. We captured students’ perceptions at a single point after a brief exposure to the concept. It remains unknown how acceptance might evolve with more prolonged or repeated exposure to digital EPAs, or after students have the opportunity to use such tools in practice. Longitudinal studies would be valuable to see if initial attitudes and intentions lead to actual usage and if they change through increased familiarity. Additionally, experimental designs (for instance, providing some students with access to a prototype digital EPA and comparing outcomes with a control group) could strengthen causal inference about the impact of digital EPAs on learning and acceptance. Despite these limitations, our cross-sectional survey provides an efficient initial evidence base that can inform future research directions and offers actionable guidance for the implementation of digital EPAs in educational practice. For educators, digital EPAs should be explicitly aligned with curricular learning objectives and assessment frameworks, so that students can clearly recognize how engagement contributes to competency progression and entrustment decisions. For developers, perceived usefulness may be strengthened by providing targeted, actionable feedback, visualizing performance data in relation to EPA levels, and ensuring that digital EPAs address authentic clinical tasks that students perceive as directly relevant to their future practice.

### Conclusions

This study provides initial empirical evidence that medical students are open to the use of digital EPAs, with acceptance primarily driven by perceived usefulness and mediated by attitude. Ease of use, while important for acceptance of analog EPAs, did not emerge as a decisive factor for the digital EPA in this conceptual, hypothetical evaluation, likely reflecting the absence of interaction with a functional system. The slightly more favorable attitudes toward the analog EPA likely reflect familiarity with traditional learning tools rather than any fundamental resistance to digitalization.

By showing that usefulness and positive attitudes are central levers for shaping students’ intent to use digital EPAs, our findings have practical implications for both developers and educators. For developers of digital EPA platforms, the message is clear: focus on features that provide tangible educational benefits and ensure the tool is reliable and adds clear value to student learning. For educators and curriculum designers, the task is to build enthusiasm and buy-in for integrating digital EPAs in a way that aligns with valued learning activities and demonstrating success stories to cultivate positive attitudes. With careful attention to demonstrating educational value and maintaining a student-centered user experience, digital EPAs have the potential to enrich competency-based medical education and better prepare students for their clinical responsibilities.

## Supplementary material

10.2196/87605 Multimedia Appendix 1 Analog and digital EPA descriptions (English).

10.2196/87605 Multimedia Appendix 2 Analoge und digitale EPA-Beschreibung (Deutsch).

10.2196/87605 Multimedia Appendix 3 Hypothesen und Fragenkatalog der TAM-Umfrage (Deutsch).

10.2196/87605 Multimedia Appendix 4 Hypotheses and questionnaire items used in the TAM survey (English).

## Abbreviations

AT: attitude (toward using the technology)
BI: behavioral intention
CFI: comparative fit index
CHERRIES: Checklist for Reporting Results of Internet E-Surveys
EPA: entrustable professional activity
LMU: Ludwig Maximilian University
OSF: Open Science Framework
PEU: perceived ease of use
PU: perceived usefulness
RMSEA: root mean square error of approximation
TAM: Technology Acceptance Model
