# Supplementary material for: Medical Students’ Acceptance of Digital Entrustable Professional Activities: Results of a Cohort Study
Source: JMIR Med Educ. 2026 May 4;12:e87605. doi: 10.2196/87605 (PMC13138705; doi:10.2196/87605)
Supplement: Multimedia Appendix 1 [file mededu-v12-e87605-s001.pdf]

EPA Description

Entrustable Professional Activities (EPAs) are a relatively new concept in medical education, designed to structure and enhance the training process for medical students and trainees. This concept was developed to bridge the gap between theoretical knowledge and its practical application in the medical profession.

Basic Concept of EPAs
EPAs are defined, essential activities within a specialty that a medical student or trainee can perform independently and responsibly once they have achieved the necessary competence. An EPA is not just a single task or skill but a combination of tasks, decisions, communication, teamwork, and ethical understanding, all of which make up a professional activity.

Why EPAs?
Traditional medical training programs often focus on imparting knowledge and skills. EPAs, on the other hand, place the learner at the center and focus on the application of knowledge in practice. They serve as a bridge between theory and clinical practice.

Structure and Levels of EPAs
Each EPA is clearly structured, with specific learning objectives and performance standards. Training and assessment are based on these defined EPAs. Typically, a learner's progress is measured in various stages:

1. **Observation and Participation**: The learner is allowed to observe and partially participate under supervision.
2. **Assisted Performance**: The learner performs the task under direct supervision.
3. **Supervised Independence**: The learner can perform the task independently but under supervision.
4. **Independence**: The learner is capable of performing the task without supervision.
5. **Teaching and Leadership**: The learner can now instruct others and teach the task independently.

Application of EPAs
EPAs are applied in various medical fields, from general clinical practice to specialized professional activities. They enable a more flexible, competency-based education tailored to the individual progress of the learners.

Benefits of EPAs

- **Practice-Oriented Learning**: EPAs promote learning through practical experience.
- **Individual Evaluation**: Learners' progress is individually assessed based on their ability to independently perform specific professional activities.
- **Flexibility**: EPAs allow for flexible learning processes adapted to each individual's pace and needs.
- **Clarity in Training**: Expectations for learners are clearly defined through EPAs.

Example of an EPA: Management of Acute Abdominal Pain
This EPA focuses on the ability of medical students and trainees to examine and assess patients with acute abdominal pain. It includes clinical evaluation, diagnosis, and the management of acute abdominal pain.

Stage 1 - Theoretical and Basic Learning:
Students first acquire foundational knowledge about the anatomy of the abdomen, physiological and pathological processes that can lead to abdominal pain, and general principles of patient examination. Additionally, students may attend and observe relevant examinations.

Stage 2 - Observation and Assisted Practice:
Under supervision, students initially perform physical examinations on patients with acute abdominal

pain. They learn how to conduct a thorough patient history and apply physical examination techniques such as inspection, palpation, percussion, and auscultation of the abdomen.

Stage 3 - Supervised Independence:
At this stage, students begin to examine patients under reduced supervision. They develop skills in differential diagnosis and learn to request and interpret relevant diagnostic tests (e.g., blood tests, ultrasound, or CT scans).

Stage 4 - Independent Practice:
Now, students can independently examine patients with acute abdominal pain and create preliminary management plans. They are capable of effectively communicating their findings and initiating appropriate referrals or treatments.

Stage 5 - Teaching and Mentoring:
In the final stage, experienced students or young doctors serve as mentors for junior colleagues. They teach examination techniques, share their experiences in diagnosing and managing patients with acute abdominal pain, and contribute to the education of the next generation of medical professionals.

Through progress in each stage of the EPA, learners gradually gain competence and confidence in examining and managing patients with acute abdominal pain. Independence increases incrementally, and only after mastering all theoretical and practical aspects is the EPA considered fully completed.

Conclusion
The concept of Entrustable Professional Activities fundamentally changes medical education by placing greater emphasis on the practical application of skills and competencies. Moreover, this concept is not tied to a strict time frame, as only the completion of all EPAs is crucial. Thus, it could be possible to complete the medical program more quickly if all EPAs are demonstrated. This offers a structured framework within which medical students and trainees can learn and demonstrate the skills required for their profession.

Digitalization of EPAs
In the context of digitalization, EPAs (Entrustable Professional Activities) have evolved. Digital EPAs within the framework of simulations are computer-assisted teaching and learning methods that complement traditional EPAs by incorporating technology. These digital tools simulate realistic medical scenarios, allowing students to gain practical experience without risking patient safety.

Role of Simulations
Simulations play a crucial role in medical education, especially in acquiring complex skills. They provide an interactive representation of real scenarios where students can apply their theoretical knowledge and practical skills. Through computer-based simulations, dynamic models of the real world and its processes are generated. These simulations allow students to practice complex skills such as visualization, classification, data interpretation, problem-solving, and experimental design.

Advantages of Digital EPAs

1. **Realistic Learning Experience**: Digital EPAs provide practical training without involving real patients.
2. **Individualized Learning**: Technology allows the learning process to be tailored to the pace and needs of each student.
3. **Safety and Ethics**: Simulations offer a safe learning environment where mistakes can be made and learned from, without endangering real patients.
4. **Efficiency**: Digital tools can contribute to a reduction in training time without compromising the quality of education.

Conclusion
Digital EPAs within the framework of simulations represent a significant innovation in medical education.

They allow medical students to gain practical experience in a low-risk environment while acquiring the competencies required for their profession. In a world where digital technologies are becoming increasingly important, digital EPAs provide an effective method to optimally prepare future doctors for their professional challenges.

Example of a Digital EPA: Management of Acute Abdominal Pain
A digital EPA could be, for example, the "Management of Acute Abdominal Pain." This skill is essential for any physician and includes various aspects, from history-taking, visual inspection, physical examination, and further patient management.

Stage 1 – Digital Theoretical Learning:
Students first learn the fundamentals of a physical examination, along with the procedures for managing such a patient through online modules and virtual simulations.

Stage 2 - Assisted Digital Performance:
In the next step, you will perform physical examinations and patient management digitally with increased guidance. Your performance and progress are digitally recorded and evaluated by the supervisor.

Stage 3 - Supervised Independence:
Once you have gained enough experience, you will conduct the examinations more independently within the digital simulation, although still with some minor assistance. Your progress will continue to be digitally tracked, and you will receive feedback.

Stage 4 - Independent Performance:
At this stage, you can manage acute abdominal pain independently within the digital simulation without assistance. Your skills and progress will be continuously documented digitally.

Stage 5 - Instruction and Teaching:
In the final stage, you can provide feedback to younger medical students on their digital EPA performance.

In this way, the theory and practice of managing acute abdominal pain would be learned and tested digitally. Progress would always be reported back to the supervisor, and through bedside teaching, the competencies learned digitally could be applied in a real environment using the previously mentioned EPA stages.

Application in the Practical Year (PJ)
At the beginning of your Practical Year, you present your supervising physician with your digital EPA portfolio (comparable to the current logbook documenting attendance). This portfolio details the skills you have already acquired and the level of supervision required for various medical activities. This allows supervising physicians to make a clear assessment of your competencies and assign you to areas where you can already work independently or where you need more experience. This contributes to a more efficient and individualized design of your Practical Year, as you can be placed in areas where your learning needs are greatest.

Conclusion
The concept of digital EPAs in medical education offers a structured and efficient method to document and assess students' progress in practical medical skills. It enables individualized and targeted training, which is highly beneficial in both the clinical phase and the Practical Year, ultimately preparing medical students optimally for their future roles as physicians.
