# Supplementary material for: Medical Students’ Acceptance of Digital Entrustable Professional Activities: Results of a Cohort Study
Source: JMIR Med Educ. 2026 May 4;12:e87605. doi: 10.2196/87605 (PMC13138705; doi:10.2196/87605)
Supplement: Multimedia Appendix 2 [file mededu-v12-e87605-s002.pdf]

**EPA-Fließtext:**

Entrustable Professional Activities (EPAs) sind ein relativ neues Konzept in der medizinischen Ausbildung, das darauf abzielt, den Ausbildungsprozess von Medizinstudierenden und angehenden Ärzt/innen zu strukturieren und zu verbessern. Dieses Konzept wurde entwickelt, um die Lücke zwischen theoretischem Wissen und praktischer Anwendung im medizinischen Beruf zu überbrücken.

**Grundkonzept von EPAs**
EPAs sind definierte, wesentliche Tätigkeiten innerhalb eines Fachgebiets, die ein Medizinstudierender oder ein Arzt/Ärztin in Ausbildung selbstständig und verantwortungsvoll ausführen kann, sobald er die dafür notwendige Kompetenz erreicht hat. Ein EPA umfasst dabei nicht nur eine einzelne Aufgabe oder Fertigkeit, sondern eine Kombination von Aufgaben, Entscheidungen, Kommunikations- und Teamfähigkeiten sowie ethischem Verständnis, die zusammen eine professionelle Tätigkeit ausmachen.

**Warum EPAs?**
Traditionelle medizinische Ausbildungsprogramme fokussieren oft auf die Vermittlung von Fachwissen und Fertigkeiten. EPAs hingegen stellen den Lernenden in den Mittelpunkt und konzentrieren sich auf die Anwendung von Wissen in der Praxis. Sie dienen als Brücke zwischen der Theorie und der klinischen Praxis.

**Strukturierung und Stufen von EPAs**
Jedes EPA ist klar strukturiert und beinhaltet spezifische Lernziele und Leistungsstandards. Die Ausbildung und Bewertung basieren auf diesen definierten EPAs. Typischerweise wird der Fortschritt eines Lernenden in verschiedenen Stufen gemessen:

1. **Beobachtung und Teilnahme**: In der ersten Stufe darf der Lernende anwesend sein, beobachten und teilweise unter Aufsicht mitwirken.
2. **Assistierte Durchführung**: Der Lernende führt die Aufgabe unter direkter Aufsicht aus.
3. **Supervisierte Unabhängigkeit**: Der Lernende kann die Aufgabe selbstständig ausführen, jedoch unter Supervision.
4. **Unabhängigkeit**: Der Lernende ist in der Lage, die Aufgabe ohne Supervision auszuführen.
5. **Lehre und Führung**: Der Lernende kann nun andere anleiten und die Aufgabe selbstständig lehren.

**Anwendung von EPAs**
EPAs finden Anwendung in verschiedenen medizinischen Bereichen, von der allgemeinen klinischen Praxis bis hin zu spezialisierten Fachärztlichentätigekiten. Sie ermöglichen eine flexiblere, kompetenzbasierte Ausbildung, die sich an den individuellen Fortschritten der Lernenden orientiert.

**Vorteile von EPAs**

- **Praxisorientiertes Lernen**: EPAs fördern das Lernen durch praktische Erfahrung.
- **Individuelle Bewertung**: Die Fortschritte der Lernenden werden individuell bewertet, basierend auf ihrer Fähigkeit, bestimmte professionelle Aktivitäten selbstständig auszuführen.
- **Flexibilität**: EPAs erlauben eine flexible Gestaltung des Lernprozesses, angepasst an die Geschwindigkeit und Bedürfnisse jedes Einzelnen.
- **Klarheit in der Ausbildung**: Durch EPAs werden die Erwartungen an die Lernenden klar definiert.

**Beispiel einer EPA: Management akuter abdomineller Schmerzen**
Diese EPA konzentriert sich auf die Fähigkeit von Medizinstudierenden und Ärzt/innen in Ausbildung, Patient/innen mit akuten abdominellen Schmerzen zu untersuchen und zu beurteilen. Sie umfasst klinische Beurteilung, Diagnosestellung, und das Management von akuten abdominellen Schmerzen.

**Stufe 1 - Theorie und Grundlagenlernen:** Zuerst erwerben die Studierenden grundlegende Kenntnisse über die Anatomie des Abdomens, die physiologischen und pathologischen Prozesse, die zu abdominellen Schmerzen führen können, und die allgemeinen Prinzipien der Patientenuntersuchung. Zudem können die Studierenden bei entsprechenden Untersuchungen anwesend sein und beobachten.

**Stufe 2 - Beobachtung und assistierte Durchführung:** Unter Aufsicht führen die Studierenden zunächst eine körperliche Untersuchung bei Patienten mit akuten abdominellen Schmerzen durch. Sie lernen, wie man eine gründliche Anamnese erhebt und die körperliche Untersuchungstechniken wie Inspektion, Palpation, Perkussion und Auskultation des Abdomens anwendet.

**Stufe 3 - Supervisierte Unabhängigkeit:** In dieser Phase beginnen die Studierenden, Patient/innen unter geringerer Aufsicht zu untersuchen. Sie entwickeln Fähigkeiten in der Differentialdiagnose und lernen, relevante diagnostische Tests (wie Bluttests, Ultraschall oder CT) anzufordern und zu interpretieren.

**Stufe 4 - Unabhängige Durchführung:** Jetzt können die Studierenden eigenständig Patienten mit akuten abdominellen Schmerzen untersuchen und vorläufige Managementpläne erstellen. Sie sind in der Lage, ihre Befunde effektiv zu kommunizieren und angemessene Überweisungen oder Behandlungen einzuleiten.

**Stufe 5 - Lehre und Anleitung:** In der letzten Phase fungieren die erfahrenen Studierenden oder jungen Ärzt/innen als Mentor/innen für jüngere Kolleg/innen. Sie lehren Untersuchungstechniken, teilen ihre Erfahrungen in der Diagnose und Behandlung von Patienten mit akuten abdominellen Schmerzen und tragen so zur Ausbildung der nächsten Generation von Medizinern bei.

Durch die Fortschritte in jeder Stufe der EPA gewinnen die Lernenden zunehmend an Kompetenz und Vertrauen in ihrer Untersuchung und Behandlung von Patient/innen mit akuten abdominellen Schmerzen. Die Selbstständigkeit steigert sich sukzessiv und erst wenn der Studierende die gesamten theoretisch praktischen Inhalte beherrscht hat er oder sie die EPA in ihrer Gesamtheit abgeschlossen.

**Fazit**
Das Konzept der Entrustable Professional Activities verändert die medizinische Ausbildung grundlegend, indem es einen stärkeren Fokus auf die praktische Anwendung von Fähigkeiten und Kompetenzen legt. Zudem ist dieses Konzept von einem strengen zeitlichen Rahmen gelöst, da nur der Erwerb aller EPAs entscheidend ist. Somit wäre es hier möglich das Studium entsprechend schneller abzuschließen, sollten alle EPAs nachgewiesen worden sein. Damit bietet es einen strukturierten Rahmen, innerhalb dessen Medizinstudierende und Ärzt/innen in Ausbildung die für ihren Beruf erforderlichen Fähigkeiten erlernen und beweisen können.

**Digitalisierung der EPAs**
Im Rahmen der Digitalisierung haben sich die EPAs weiterentwickelt. Digitale EPAs im Kontext von Simulationen sind computergestützte Lehr- und Lernmethoden, die die traditionellen EPAs durch den Einsatz von Technologie ergänzen. Diese digitalen Tools simulieren realistische medizinische Szenarien, in denen Studierende praktische Erfahrungen sammeln können, ohne Patient/innen zu gefährden.

**Rolle von Simulationen**
Simulationen spielen eine entscheidende Rolle in der medizinischen Ausbildung, besonders beim Erwerb komplexer Fähigkeiten. Sie bieten eine interaktive Darstellung realer Szenarien, in denen Studierende ihr theoretisches Wissen und ihre praktischen Fähigkeiten anwenden können. Durch computerbasierte Simulationen werden dynamische Modelle der realen Welt und ihrer Prozesse erzeugt. Sie ermöglichen es den Studierenden, komplexe Fähigkeiten wie Visualisierung, Klassifikation, Dateninterpretation, Problemlösung und experimentelles Design zu üben.

**Vorteile digitaler EPAs**

1. **Realitätsnahe Lernerfahrung**: Digitale EPAs ermöglichen eine praxisnahe Ausbildung, ohne reale Patienten zu involvieren.
2. **Individuelles Lernen**: Die Technologie erlaubt eine individuelle Anpassung des Lernprozesses an das Tempo und die Bedürfnisse jedes Studierenden.
3. **Sicherheit und Ethik**: Simulationen bieten eine sichere Lernumgebung, in denen Fehler gemacht und daraus gelernt werden kann, ohne reale Patienten zu gefährden.

4. **Effizienz**: Digitale Tools können zu einer Verkürzung der Ausbildungszeit beitragen, ohne die Qualität der Ausbildung zu beeinträchtigen.

**Fazit**
Digitale EPAs im Rahmen von Simulationen stellen eine bedeutende Innovation in der medizinischen Ausbildung dar. Sie ermöglichen es Medizinstudierenden, in einer risikoarmen Umgebung praktische Erfahrungen zu sammeln und gleichzeitig die für ihren Beruf erforderlichen Kompetenzen zu erwerben. In einer Welt, in der digitale Technologien immer mehr an Bedeutung gewinnen, bieten digitale EPAs eine effektive Methode, um zukünftige Ärzt/innen optimal auf ihre beruflichen Herausforderungen vorzubereiten.

**Beispiel einer digitalen EPA: Management akuter abdomineller Schmerzen**
Eine digitale EPA könnte beispielsweise das "Management akuter abdomineller Schmerzen" sein. Diese Fertigkeit ist essentiell für jeden Mediziner und umfasst verschiedene Aspekte, von der Anamnese, visuellen Inspektion, der körperlichen Untersuchung und des weiteren Patientenmanagements.

**Stufe 1 – Digitales theoretisches Lernen**: Zunächst lernen die Studierenden die Grundlagen einer körperlichen Untersuchung, sowie das weitere Verfahren mit solch einem/r Patient/in durch Online-Module und virtuelle Simulationen.

**Stufe 2 - Assistierte digitale Durchführung**: Im nächsten Schritt führen Sie unter verstärkter Hilfestellung selbst körperliche Untersuchungen und Patient/Innen Management digital durch. Ihre Leistungen und Fortschritte werden digital erfasst und durch den Supervisor bewertet.

**Stufe 3 - Supervisierte Unabhängigkeit**: Sobald Sie genügend Erfahrung gesammelt haben, führen Sie die Untersuchungen im Rahmen der digitalen Simulation selbstständiger durch, aber immer noch unter kleineren Hilfestellungen. Weiterhin wird ihr Fortschritt digital erfasst und sie erhalten Feedback.

**Stufe 4 - Unabhängige Durchführung**: Jetzt sind Sie in der Lage, eigenständig und ohne Hilfestellungen das Management von akuten abdominellen Schmerzen in der digitalen Simulation durchzuführen. Ihre Fähigkeiten und Fortschritte werden kontinuierlich digital dokumentiert.

**Stufe 5 - Anleitung und Lehre**: In der letzten Stufe können Sie anderen, jüngeren Medizinstudierenden Feedback auf deren digitale EPA Leistung geben an.

Insgesamt würde so die Theorie und Praxis des Managements von akuten abdominellen Schmerzen auf digitale Weise erlernt und erprobt werden. Der Fortschritt würde stets dem Supervisor zurückgemeldet werden und durch Bed-Side Teaching könnten die digital erlernten Kompetenzen in realer Umgebung erprobt werden anhand der zuvor genannten EPA-Abstufung.

**Anwendung im Praktischen Jahr (PJ)**
Zu Beginn Ihres Praktischen Jahres präsentieren Sie Ihrem betreuenden Arzt/Ärztin Ihr digitales EPA-Portfolio. (Vergleichbar mit dem aktuellen Logbuch, das die Anwesenheit dokumentiert) Dieses zeigt detailliert auf, welche Fähigkeiten Sie bereits erworben haben und auf welchem Niveau der Supervision Sie sich in verschiedenen medizinischen Aktivitäten befinden.
Dies ermöglicht den betreuenden Ärzt/innen, eine klare Einschätzung Ihrer Kompetenzen vorzunehmen und Sie gezielter in Bereichen einzusetzen, in denen Sie bereits selbstständig arbeiten können oder in denen Sie noch weitere Erfahrung sammeln müssen. Dies trägt zu einer effizienteren und individuelleren Gestaltung Ihres Praktischen Jahres bei, da Sie in den Bereichen eingesetzt werden können, in denen Ihr Lernbedarf am größten ist.

**Fazit**
Das Konzept der digitalen EPAs im Medizinstudium bietet eine strukturierte und effiziente Methode, um den Fortschritt der Studierenden in praktischen medizinischen Fähigkeiten zu dokumentieren und zu bewerten. Es ermöglicht eine individuelle und zielgerichtete Ausbildung, die sowohl im klinischen

Abschnitt als auch im Praktischen Jahr von großem Nutzen ist. Dadurch werden Medizinstudierende optimal auf ihre zukünftigen Aufgaben als Ärzt/innen vorbereitet.
