# Supplementary material for: Medical Students’ Acceptance of Digital Entrustable Professional Activities: Results of a Cohort Study
Source: JMIR Med Educ. 2026 May 4;12:e87605. doi: 10.2196/87605 (PMC13138705; doi:10.2196/87605)
Supplement: Multimedia Appendix 3 [file mededu-v12-e87605-s003.pdf]

Hypothesen:

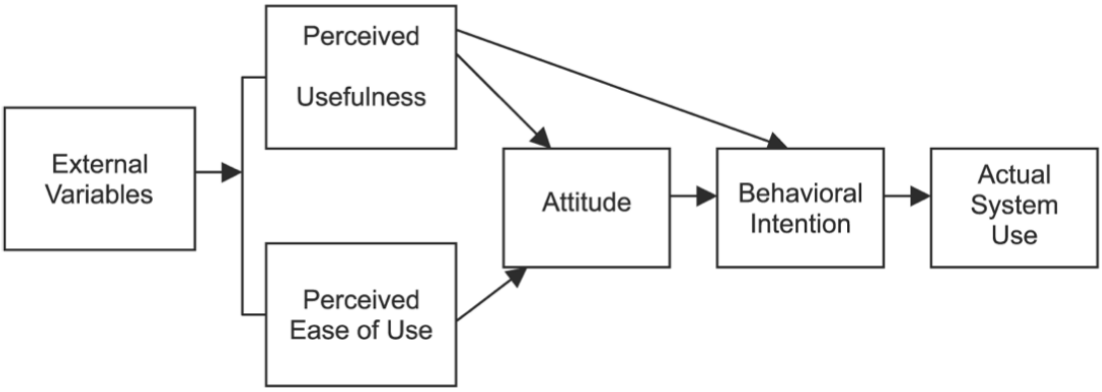

Figure 1: Technology Acceptance Model

Wahrgenommene Nützlichkeit (PU)
Hypothese 1:
Je nützlicher Medizinstudierenden digitale EPAs in Simulationen empfinden, desto höher ist ihre Bereitschaft, diese Technologie im Rahmen ihres Studiums zu nutzen.

Wahrgenommene Benutzerfreundlichkeit (PEU)
Hypothese 2:
Je einfacher Medizinstudierende die Bedienung digitaler EPAs in medizinischen Simulationen empfinden, desto größer ist ihre Bereitschaft, diese Technologie zu nutzen.

Einstellung (AT)
Hypothese 3:
Je positiver die Einstellung von Medizinstudierenden gegenüber digitalen EPAs in medizinischen Simulationen ist, desto eher werden sie diese Technologie nutzen.

Konstrukt
Wahrgenommene Nützlichkeit (PU)

Operationale Definition
Die wahrgenommene Nützlichkeit spiegelt die Wahrnehmung der Studierenden wider, ob der Einsatz digitaler EPAs in einer simulierten Umgebung ihre Leistung verbessern wird.

Gemessene Elemente
PU1: Eine digitale EPA wird meine Lernleistung verbessern.
PU2: Eine digitale EPA könnte das Erlernen von Kursinhalten erleichtern.
PU3: Eine digitale EPA wird die Effektivität des Lernens steigern.
PU4: Ich finde digitale EPAs nützlich.

Konstrukt
Wahrgenommene Benutzerfreundlichkeit (PEU)

Operationale Definition
Die wahrgenommene Benutzerfreundlichkeit bezieht sich auf die Wahrnehmung eines Studierenden, dass die Verwendung digitaler EPAs für das Erlernen medizinischer Fähigkeiten im Rahmen des Studiums nur minimalen Aufwand erfordern wird.

Gemessene Elemente
PEU1: Es fällt mir leicht zu lernen wie man die digitale EPA nutzt.
PEU2: Es ist einfach im Umgang mit der digitalen EPA vertraut zu werden.
PEU3: Meine Interaktion mit der digitalen EPA ist klar.
PEU4: Meine Interaktion mit der digitalen EPA ist verständlich.

Konstrukt
Einstellung (AT)

Operationale Definition
Einstellung bezieht sich auf das Urteil der Studierenden darüber, ob die Verwendung digitaler EPAs für sie von Vorteil ist.

Gemessene Elemente
AT1: Mit der digitalen EPA zu lernen, ist eine gute Idee.
AT2: Ich stehe der Verwendung der digitalen EPA positiv gegenüber.
AT3: Ich glaube, dass die digitale EPA mir hilft, mich stärker in das Lernen einzubinden.
AT4: Ich befürworte im Allgemeinen den Einsatz der digitalen EPA für das Lernen.
AT5: Ich glaube, dass es eine gute Idee ist, digitale EPAs für meine zukünftigen Kurse zu verwenden.

Konstrukt:
Verhaltensabsicht (BI)

Operationale Definition:
Die Verhaltensabsicht bezieht sich auf die theoretische Absicht der Studierenden, digitale EPAs für ihr Studium in der klinischen Phase des Medizinstudiums zu nutzen, falls diese angeboten würden.

Gemessene Elemente
BI1: Ich würde das mobile digitale Spiel häufig nutzen, um medizinische Fähigkeiten zu üben.
BI2: Ich würde die digitale EPA intensiv nutzen.
BI3: Ich würde digitale EPAs während des Semesters verwenden und auch im nächsten Semester.
BI4: Ich würde digitale EPAs so oft wie möglich wiederholt nutzen.
