# Supplementary material for: Medical Students’ Acceptance of Digital Entrustable Professional Activities: Results of a Cohort Study
Source: JMIR Med Educ. 2026 May 4;12:e87605. doi: 10.2196/87605 (PMC13138705; doi:10.2196/87605)
Supplement: Multimedia Appendix 4 [file mededu-v12-e87605-s004.pdf]

Hypotheses

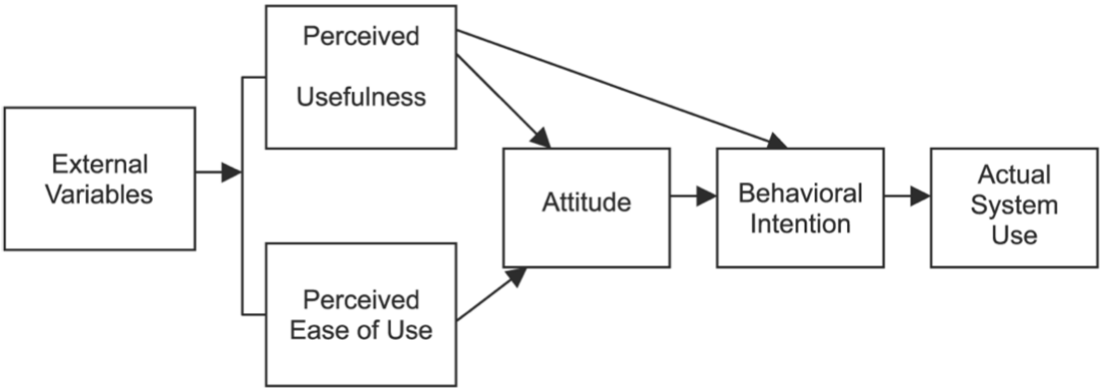

Figure 1: Technology Acceptance Model

Perceived Usefulness (PU)
Hypothesis 1:
The more useful medical students find digital EPAs in medical simulations, the higher their behavioural intention to use this technology as part of their studies.

Construct: Perceived Ease of Use (PEU)
Hypothesis 2:
The easier medical students find the operation of digital EPAs in medical simulations, the greater their behavioural intention to use this technology.

Construct: Attitude (AT)
Hypothesis 3:
The more positive the attitude of medical students towards digital EPAs in medical simulations, the higher their behavioural intention to use this technology.

Construct
Perceived Usefulness (PU)

Operational Definition
Perceived usefulness reflects students' perception on whether the use of digital EPAs in a simulated environment will enhance their performance.

Measured Items
PU1: The digital EPA will improve my learning performance.
PU2: The digital EPA could make it easier to study course content.
PU3: The digital EPA will enhance the effectiveness of learning.
PU4: I find the digital EPA in the simulation environment useful.

Construct
Perceived Ease of Use (PEU)

Operational Definition
Perceived ease of use refers to a student's perception that using digital EPAs for learning skills during their medical studies will require minimal effort.

Measured Items
PEU1: Learning how to use the digital EPA is easy for me.
PEU2: It is easy to become skillful in using the digital EPA.
PEU3: My interaction with the digital EPA is clear.
PEU4: My interaction with the digital EPA is understandable.

Construct
Attitude (AT)

Operational Definition
Attitude refers to student's judgement on whether the use of digital EPAs is beneficial to them.
Measured Items
AT1: Studying using the digital EPA is a good idea.
AT2: I feel positive towards the use of the digital EPA.
AT3: I believe that the digital EPA helps me to be more engaged in learning.
AT4: I generally favour the use of the digital EPA for learning.
AT5: I believe that it is a good idea for me to use digital EPA for my future coursework.

Construct
Behavioral Intention (BI)

Operational Definition
Behavioural intention refers to a students' intention to theoretically use digital EPAs for their studies during the clinical phase of medical school if they were 2ob e offered.

Measured Items
BI1: I would frequently use the mobile digital game to practice medical skills.
BI2: I would to use the digital EPA heavily.
BI3: I would use digital EPAs throughout my semester and continue throughout the next.
BI4: I would repetitively use digital EPAs as often as possible.
